# Strain Effects in Twisted Spiral Antimonene

**DOI:** 10.1002/advs.202301326

**Published:** 2023-04-24

**Authors:** Ding‐Ming Huang, Xu Wu, Kai Chang, Hao Hu, Ye‐Liang Wang, H. Q. Xu, Jian‐Jun Zhang

**Affiliations:** ^1^ Beijing Academy of Quantum Information Sciences Beijing 100193 China; ^2^ Beijing Key Laboratory of Quantum Devices Key Laboratory for the Physics and Chemistry of Nanodevices and School of Electronics Peking University Beijing 100871 China; ^3^ National Laboratory for Condensed Matter Physics and Institute of Physics Chinese Academy of Sciences Beijing 100190 China; ^4^ MIIT Key Laboratory for Low‐Dimensional Quantum Structure and Devices School of Integrated Circuits and Electronics Beijing Institute of Technology Beijing 100081 China; ^5^ Frontier Institute of Science and Technology Xi'an Jiaotong University Xi'an 710054 China

**Keywords:** Antimonene, helical dislocation, spiral, strain effect, vdW layered material

## Abstract

Van der Waals (vdW) layered materials exhibit fruitful novel physical properties. The energy band of such materials depends strongly on their structures, and a tremendous variation in their physical properties can be deduced from a tiny change in inter‐layer spacing, twist angle, or in‐plane strain. In this work, a kind of vdW layered material of spiral antimonene is constructed, and the strain effects in the material are studied. The spiral antimonene is grown on a germanium (Ge) substrate and is induced by a helical dislocation penetrating through few atomic‐layers of antimonene (*β*‐phase). The as‐grown spiral is intrinsically strained, and the lattice distortion is found to be pinned around the dislocation. Both spontaneous inter‐layer twist and in‐plane anisotropic strain are observed in scanning tunneling microscope (STM) measurements. The strain in the spiral antimonene can be significantly modified by STM tip interaction, leading to a variation in the surface electronic density of states (DOS) and a large modification in the work function of up to a few hundreds of millielectron‐volts (meV). Those strain effects are expected to have potential applications in building up novel piezoelectric devices.

## Graphical abstract

Antimonene spirals are epitaxially grown on a germanium substrate. Owing to the presence of a helical dislocation, the as‐grown spiral is anisotropically strained. The strain in the spiral can be changed by scanning tunneling microscopy tip manipulation, resulting in modulations of the electronic density of states and the work function.

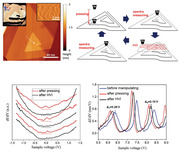

## 1 Introduction

Two‐dimensional (2D) van der Waals (vdW) layered materials are widely studied in the past two decades.^[^ ^1^, ^2^, ^3^, ^4^, ^5^, ^6^, ^7^, ^8^, ^10^, ^11^, ^12^, ^13^, ^14^, ^15^, ^16^, ^17^, ^18^ ^]^ These materials are vdW stackings of atomic layers of 2D covalently bonded materials, and their electronic property significantly relies on the inter‐layer coupling. A certain setting of the inter‐layer coupling or stacking order in such materials may emerge exotic physical properties. Twisted graphene stacking layers are of a well‐known case, which exhibits superconductivity,^[^ ^1^ ^]^ quantized anomalous Hall states,^[^ ^2^ ^]^ or electronic nematicity^[^ ^3^ ^]^ under different stacking orders. A tiny change in the inter‐layer coupling can lead to a significant change in the band structure, such as that the flat band can be induced by the inter‐layer compression.^[^ ^4^, ^5^ ^]^ The in‐plane strain, either a global strain or a local strain induced by scanning tunneling microscope (STM) tip interaction, in the stacking layers also has a large impact on the band structure^[^ ^6^, ^7^, ^8^ ^]^ and thus electronic properties. As an example, the shift of van‐Hove singularities in graphene stacking layers induced by a local strain has been observed.^[^ ^7^ ^]^ Although the strain effects are widely studied on numerous 2D vdW layers systems, relevant research on spiral structures^[^ ^9^ ^]^ is rarely reported. These spirals are of a kind of vdW layered materials with a helical dislocation penetrated through them. There may exist novel strain effects, as the interaction between layers is affected by the helical dislocation.

Recently, antimonene (*β*‐phase) stacking layers have attracted great research interest. Antimonene is a buckled honeycomb 2D material of the antimony (Sb). It is expected to have applications for 2D photoelectric devices, owing to the semiconducting feature of the free‐standing antimonene.^[^ ^10^ ^]^ Exotic properties were observed in different antimonene stacking layers, such as semiconductor‐semimetal transitions,^[^ ^10^, ^11^ ^]^ topological surface states,^[^ ^12^, ^13^ ^]^ and flat bands.^[^ ^14^, ^15^ ^]^ Few‐layers antimonene is also predicted to exhibit fruitful strain effects, such as a band gap modulation induced by inter‐layer compression,^[^ ^11^ ^]^ and the quantum spin Hall state induced by in‐plane strain.^[^ ^16^, ^17^, ^18^ ^]^ Thus, spiral antimonene is a proper candidate for studying the strain effects on spirals.

Here, we report the epitaxial growth of spiral antimonene on germanium (Ge) substrate by molecular beam epitaxy (MBE). Inter‐layer twist forms in the spiral, as it is revealed by the moiré pattern. Quasi‐particle interference (QPI) measurements show that the as‐grown spiral is anisotropically strained. The strain in the spiral can also be manipulated by STM tip, as a tip induced large variation in the surface electronic density of states (DOS) and the work function are found. These strain‐dependent properties are expected to have potential applications in constructing novel piezoelectric devices.

## 2 Results

**Figure 1:**
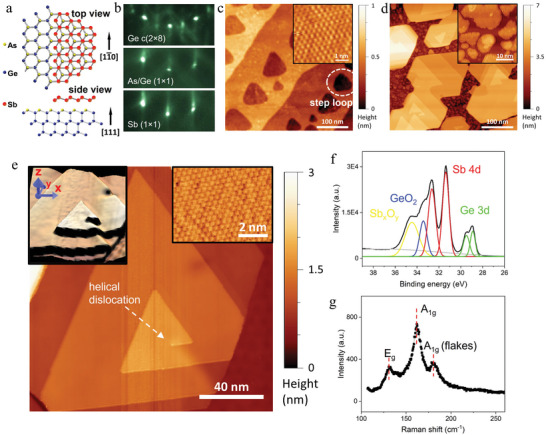
Spiral antimonene on a Ge (111) substrate. a) Schematic of the antimonene (partially covered) on an As/Ge surface. b) RHEED patterns of the Ge (111) c(2 × 8) surface, the As/Ge (1 × 1) surface and the spiral antimonene surface, respectively. Surface reconstruction is vanished once the substrate is fully covered with As. The “ × 1” feature of the RHEED pattern seen in the lowest panel of b) indicates that all antimonene spirals have the same crystal orientation. c) STM topographic image of the As/Ge surface with “step loops” (taken at 1.03 V and 65 pA). The inset shows an atomic resolution image of the top As atomic layer. d) STM topographic image of antimonene spirals (taken at 1.28 V and 95 pA). The inset shows antimonene flakes between spirals. e) STM image of a spiral (taken at −1.86 V and −150 pA), where a helical dislocation is marked by an arrow. Top‐left inset: 3D view. Top‐right inset: atomic resolution STM image of the antimonene buckled honeycomb lattice. f) XPS data of the spirals. Two main peaks (red fitted curves) correspond to Sb 4d orbits in antimonene, with binding energies of 31.4 eV and 32.6 eV, respectively. g) Raman spectrum of the spirals. Three peaks from left to right arise from the *E*_g_ and *A*_1g_ modes of the spirals and the *A*_1g_ mode of the antimonene flakes, respectively. The Raman shift is accordant with *β*‐phase antimonene.

The spiral antimonene is grown on a Ge (111) substrate. Before Sb deposition, a monolayer of arsenic (As) is deposited on the Ge substrate at 920 K to form an unreconstructed As‐terminated (1 × 1) surface. **Figure** **1**a is the schematics showing the top and side view of antimonene formed on As/Ge surface. The left part in the top panel of Figure 1a is uncovered with antimonene to make the bottom As and Ge atoms clearly visible. Each As atom has five valence electrons and three of them are bonded to Ge in the second layer, leaving two electrons forming a lone‐pair on the surface.^[^ ^19^ ^]^ Thus, a surface with no dangling bonds is obtained, which is an ideal platform for vdW epitaxy of antimonene. The surface reconstruction during As covering is monitored by in situ reflection high‐energy electron diffraction (RHEED), which shows the transition from the “ × 2” pattern of Ge (111) c (2 × 8) reconstruction to “ × 1” unreconstructed pattern (Figure 1b). The distribution of atomic steps can be controlled by As beam flux during growth. Bi‐layer deep “step loops” are formed at a temperature of 920 K and an effective As flux pressure of 8.0 × 10^−6^ mbar. An STM image of “step loops” is shown in Figure 1c. The (1 × 1) surface is confirmed by the atomic resolution STM image shown in the inset of Figure 1c. Such bi‐layer deep and dangling‐bond free “step loops” are used to grow spiral antimonene.

Sb is deposited in two steps. At first, 0.05 ML of Sb is deposited at 280 K to form Sb clusters. The sample is then heated to 400 K and exposed to Sb beam flux for 10 min under an effective Sb pressure of 3.0 × 10^−7^ mbar. Antimonene spirals are observed on the As/Ge surface as helical dislocation is formed inside “step loops”. The formation of dislocation is discussed in Section SI (Supporting Information). The STM topographic images after Sb deposition are shown in Figure 1d,e with different scales, where the helical dislocation is indicated by an arrow. The left‐top inset of Figure 1e shows the 3D view of the spiral. The measured step height on the spiral is 4.0 Å, which is equal to one atomic layer of the antimonene. The average thickness of spirals grown in this work is 10 atomic layers. The density of helical dislocations is about 50 µm^−2^, which is similar to the density of surface “step loops”. The RHEED diffraction pattern after Sb deposition is displayed in Figure 1b. The clear (1 × 1) pattern indicates that all spirals have the same crystal orientation. The lattice constant of the antimonene is measured as 4.0 Å, indicating that it is under a large compressive strain (3% smaller than the theoretical value 4.12 Å of free standing antimonene^[^ ^20^ ^]^). Such a large strain limits their lateral growth. The lateral growth continues only after the formation of helical dislocation, which releases the strain energy. This also explains why the lateral size of the spirals can reach to ≈100 nm, while the diameter of antimonene flakes between spirals is <30 nm, as shown in inset of Figure 1d.

The as‐grown antimonene is characterized by in situ STM, ex situ X‐ray photoelectron spectroscopy (XPS) and Raman spectrum. The atomic resolution STM measurements show a buckled honeycomb lattice (the inset of Figure 1e). The XPS data is shown in Figure 1f, where the two main peaks (red) seen at 31.4 and 32.6 eV are identified to be from Sb 4d orbits.^[^ ^21^ ^]^ The peaks on the lower energy side of the main peaks are from Ge 3d orbits in the substrate (green), while the peaks on the higher energy side of the main peaks are from GeO_2_ (blue) and Sb_x_O_y_ (yellow) due to oxidization in air.^[^ ^22^, ^23^ ^]^ Figure 1g shows the Raman spectrum of the spirals. The in‐plane vibration mode *E*_g_ and out‐of‐plane vibration mode A_1g_ of antimonene are observed. The Raman peak at 179.7 cm^−1^ is the *A*_1g_ mode for single layer antimonene flakes. The peaks at 131.5 and 161.6 cm^−1^ are *E*_g_ and *A*_1g_ modes of the spirals, which have a redshift of the vibration frequency due to the stacking of 10 layers.^[^ ^24^ ^]^ Both the XPS and the Raman spectrum are measured with a light spot of a few micrometers in diameter. The spectra are average results over thousands of spirals.

**Figure 2:**
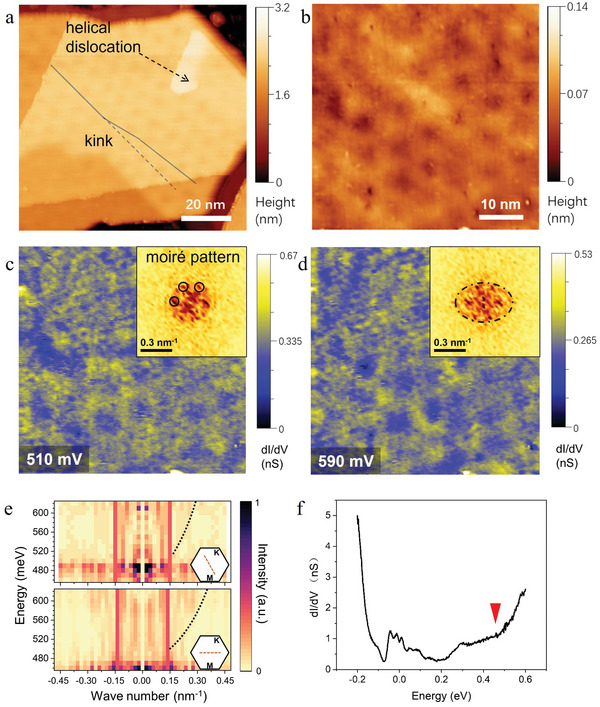
Strain in an antimonene spiral. a) Moiré lattice on the spiral. The solid line illustrates a kink on moiré pattern, corresponding to a lattice distortion near the helical dislocation. b) Topographic image of the QPI measuring region (taken at +510 mV and +335 pA). c,d) dI/dV spatial maps at sample voltages of +510 mV and +590 mV, respectively (with a 34 mV modulation and tunneling current of +335 pA). Insets: the corresponding FFT images. The oval contour ring in d) indicates the electron scattering is anisotropic. e) QPI dispersion. Dashed lines illustrate the energy level versus the wave number of intra‐band scattering vectors in two Γ–K directions. f) dI/dV spectrum on the QPI measuring region (taken at +470 mV, +335 pA, and a 4 mV modulation). The red triangle marks the energy position of 465 meV at which the DOS turns to a quicker increase.

As shown in **Figure** **2**a, we see a moiré pattern on the spiral antimonene, indicating the existence of an inter‐layer twist. The moiré periods are from 7 to 10 nm in different spirals, corresponding to interlayer twisted angles of 2.3^o^ to 3.3^o^. The difference in twisted angle may originate from the random size of “step loops” on the substrate. The moiré periods of some spirals show also a small deformation, as illustrated by the solid line in Figure 2a. Such a distortion is attributed to local strain caused by helical dislocation.

In‐plane strain is able to modify the elastic scattering of surface electrons, which could result in symmetry breaking in QPI.^[^ ^25^, ^26^ ^]^ Spatial dI/dV maps are performed at low temperature of 10 K to acquire QPI signal on spirals. The STM topography of a mapping region next to a helical dislocation is shown in Figure 2b. In order to intuitively demonstrate the anisotropic QPI signal, dI/dV maps taken at sample bias of +510 and +590 mV are selected to show (Figures. 2c,d, respectively). Both are performed under condition of 34 mV modulated amplitude and constant tunneling current of +335 pA. The insets show the 2D fast Fourier transform (FFT) of the dI/dV maps. The six‐fold symmetrical points in FFT images are signals from the moiré pattern (marked in inset of Figure 2c), which are invariant as the sample voltage increases. There exists a contour ring encircling the Brillouin zone center in the FFT images (dashed line in inset of Figure 2d), which is attributed to an intra‐band scattering in a conduction band, as the diameter of the contour ring expands when the sample voltage increases. An oval shape of the contour ring indicates that the electron scattering is biaxially anisotropic, which is different from the six‐fold symmetry of intrinsic antimonene stacking layers. The QPI dispersions are obtained by integrating the linecuts in FFT of dI/dV maps, and the bias voltage of the dI/dV maps is ramped from +460 to +620 mV (Figure 2e). The electron‐pocket‐like energy dependence of the scattering vector (dashed lines in Figure 2e) indicates a band minimum at 465 ± 10 meV. The detailed data processing can be found in Section S‐II (Supporting Information). The surface DOS in the QPI measuring region is characterized by dI/dV spectrum measurement (Figure 2f). We speculate that the DOS spike at 465 meV is caused by the band minimum, which is according to the data of QPI. The QPI dispersion is clearly anisotropic in two Γ–K directions (Figure 2e), and the ratio of electron effective mass in these directions is calculated as 1.6:1 (S‐II, Supporting Information). Such anisotropy is a strong evidence of strain, which is pinned around the helical dislocation and spreads over about 100 nm in lateral directions.

**Figure 3:**
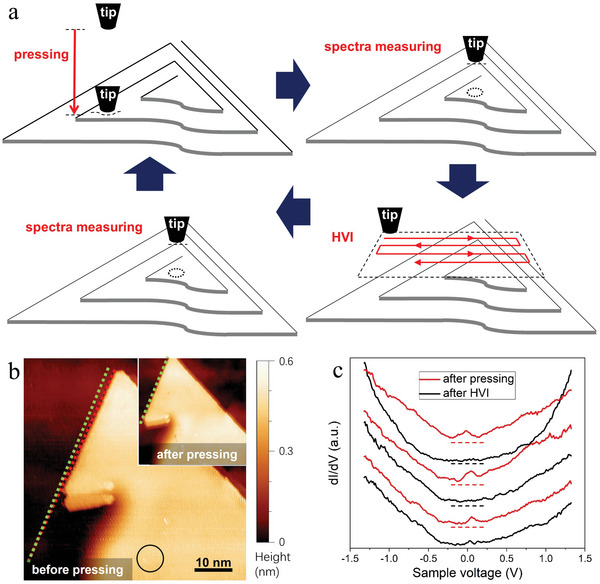
DOS modification induced by STM tip manipulating. a) Schematic diagram of the tip manipulating and the spectra measuring. b) The twist of the top layer after tip‐pressing. Twist of 1.0^o^±0.3^o^ is illustrated by the dashed line in STM image (taken at −1.3 and −102 pA). The pressing position is marked by circle. c) The DOS variation induced by tip manipulating. dI/dV spectra are measured on the spiral (−1.3 V, −102 pA, 15 mV modulation), during three cycles of tip pressing and HVI. A broad peak at 0 bias is observed after pressing (red) and disappeared after HVI (black).

Previous studies reveal that a STM tip is able to manipulate the strain in graphene stacking layers, leading to variation in physical properties.^[^ ^7^, ^8^ ^]^ Strain in spiral antimonene can also be manipulated by a STM tip. We demonstrate that the surface DOS and the work function can be tuned by strain via performing “tip pressing” and “high voltage imaging” (HVI) on the spirals. **Figure** **3**a schematically shows the processes of tip manipulation and property characterization. For “tip pressing”, we directly reduce the tip height to the spiral surface. The strong repulsive force between the tip and the spiral induces distortion around the helical dislocation, such as a top layer twist of 1.0^o^±0.3^o^ (Figure 3b). Determination of the twist angle and the error analysis are detailed in Section S‐III (Supporting Information). HVI is a conventional imaging process, with a high sample bias and a high tunneling current. Details of the tip pressing and the HVI are described in the Experimental Section. Characterizations of the DOS and the work function are performed next to the pressing position (with ≈30 nm in lateral distance).

Tip pressing and HVI are iteratively performed on a spiral. The surface DOS is characterized by dI/dV spectra. Six dI/dV spectra are displayed in Figure 3c, which are measured in the order as illustrated in Figure 3a. A broad peak near the Fermi‐level is observed after tip pressing (red), the feature of the curves is similar to the spectrum shown in Figure 2f. In contrast, the broad peak vanishes after HVI (black), and the feature is similar to the semi‐metal few‐layers antimonene in previous reports.^[^ ^27^ ^]^ All of the dI/dV spectra are measured under the same tunneling condition of −1.3 V, −102 pA and a modulation amplitude of 15 mV.

**Figure 4:**
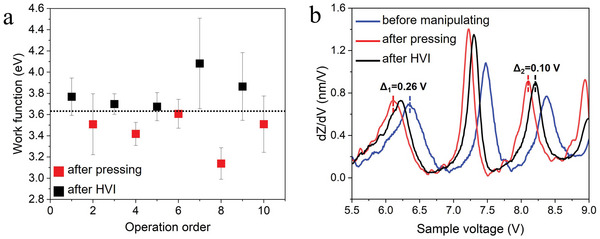
Work function variation induced by tip manipulating. a) Work function variation characterized by I–Z spectra. The X‐coordinate is the operation order, tip manipulating of HVT, and “tip pressing” are represented by odd and even numbers, respectively. Error bar is the RMS error from 6 times of measurements. b) Work function variation characterized by dZ/dV spectra. The energy shifts of the “Gundlach oscillation” peaks indicate work function variations of −0.26 ± 0.01 eV and +0.1 ± 0.01 eV, respectively.

The work function variation on a spiral is shown in **Figure** **4**a. The work function values are obtained by tunnel‐junction‐apparent‐barrier‐height in I–Z spectra. Detailed data processing and the error analysis are described in Section S‐IV (Supporting Information). The work function decreases by hundreds of meV after tip pressing, and gets back after HVI. Each data point in Figure 4a is an average result from 6 times of measurements, while the error bar is the root‐mean‐square (RMS) error. In addition, dZ/dV spectra are also measured to determine the work function variation on a spiral (Figure 4b). The “Gundlach oscillation” peaks in dZ/dV spectrum correspond to the field resonance states in tunnel junction.^[^ ^28^ ^]^ The energy shift of the peaks is equal to the difference of work function, as long as the electric field in tunnel junction is invariant in measurements.^[^ ^29^ ^]^ The energy shifts induced by tip pressing and HVI in Figure 4b are −0.26 ± 0.01 eV and +0.1 ± 0.01 eV, respectively, which further demonstrates the hundreds of meV of variation in the work function. The dZ/dV spectra are obtained by numerical differentiation of the Z–V spectra. More details of the work function measurements can be found in Section S‐IV (Supporting Information).

## 3 Discussion

The modifications of DOS and work function are considered to originate from the variation in strain. The strain induced by tip pressing can reside in a spiral, as the distortion can be pinned around the helical dislocation. On the other hand, the HVI generates a strong electric field between the tip and the spiral, and the spiral tends to release the pressing‐induced strain under such perturbation. The distortion in spiral can be classified into in‐plane and inter‐layer distortion, respectively. The in‐plane distortion has been observed as the twist of the top layer (Figure 3b). But there is lack of direct observation of the inter‐layer distortion, as such sub‐Angstrom change in height is too small to confirm by STM. We speculate that the inter‐layer distortion has the main contribution to the variations in DOS and work function. A suppression of the inter‐layer coupling, which is caused by an inter‐layer distortion, may lead the top layer of the spiral to become nearly freestanding. The band structure of the nearly freestanding top layer should be similar to the semiconducting antimonene;^[^ ^10^, ^11^ ^]^ that is why the electronic states near the Fermi‐level is able to be manipulated by STM tip (Figure 3c). The work function also significantly depends on the inter‐layer coupling; a change of several hundred meV in work function can be achieved by layers stacking.^[^ ^30^, ^31^, ^32^ ^]^ In the other hand, the work function of few‐atomic‐layers films is affected by quantum well states, which is significantly relied on the material thickness.^[^ ^33^ ^]^ Although the tip induced inter‐layer distortion is sub‐Angstrom, the change in inter‐layer coupling still may lead tremendous changes in the “effective thickness” and the work function. An indirect evidence of the tip‐induced inter‐layer distortion is discussed in Section S‐V (Supporting Information). In order to confirm that the variations in DOS and work function are caused by the STM tip manipulating, but not measurement error from local‐states on moiré lattice, local electronic states and local work function are studied in detail for comparison in Section S‐VI (Supporting Information).

## 4 Conclusion

In summary, we demonstrate the epitaxial growth of the spiral antimonene on Ge (111) substrate, and the strain related electronic properties of the spiral antimonene are investigated at nanoscale. Intrinsic in‐plane anisotropic strain is observed in the spiral, as the helical dislocation in the spiral is a natural pinning center for lattice distortion. The distortion induced by STM tip interaction is able to reside in the spiral, lending to a change of strain condition. The surface DOS and the work function of the spiral antimonene are relied on the strain, which can be repeatedly manipulated by STM tip interaction. Such strain‐dependent modifications of DOS and work function are expected to have potential applications in novel piezoelectric devices or contact technique for nanodevices. The spiral also provides an ideal platform to study the strain effects around the lattice dislocation.

## 5 Experimental Section

### Growth

The MBE was interconnected by two growth chambers, one for Ge deposition and the other for As, Sb deposition. At first, a Ge (111) substrate was heated at 890 K for 6 min in the Ge‐growth chamber to remove the native oxidation. Then, the temperature was ramped down to 590 K for deposition of a 50 nm Ge buffer layer, and the growth rate was 0.5 Å s^−1^. A flat Ge surface was achieved after an annealing process at 690 K for 5 min. The atomic steps on such surface were arranged in parallel array. After the buffer layer, the sample was transported to the As‐Sb‐growth chamber, and the background pressure was <3.4 × 10^−9^ mbar during the transport. The sample was exposed to an As_4_ molecule flux with an effective pressure of 8.0 × 10^−6^ mbar (measured by in situ beam flux monitor), once it was loaded in the As‐Sb‐growth chamber. The As_4_ flux was evaporated from an elementary cracker source. Then, the substrate temperature was rapidly ramped up to 920 K and held for 10 min under the As_4_ flux. The Ge (111) surface was passivated by a single atomic layer of As atoms under such condition, and the “step loops” were formed at the same time. After the As passivation, we shut down the As_4_ flux and ramped the temperature down to 280 K, in preparation for the followed Sb deposition. Sb was evaporated from an elementary cracker source. The Sb deposition was consisted of two steps: 0.05 ML deposition of the nucleating layer at 280 K, followed by the growth of spiral antimonene at 400 K. The effective pressure of the Sb flux was controlled at 3.0 × 10^−7^ mbar to achieve the growth of spirals.

### STM Experiments

The STM chamber was connected to the MBE chamber in vacuum. All of the STM measurements were performed under the low temperature of 10 K, and the temperature was held by a cryostat. The STM tip was a tungsten (W) tip, which was obtained by electrochemical corrosion. The tip was treated and spectroscopically characterized on the Au (111) surface before experiments, to acquire a standard tip for spectroscopic measurements. The dZ/dV spectra were obtained by numerical differentiation of the Z–V spectra. The Z–V spectra were measured by ramping the sample bias, in the condition of the tunneling current was fixed. The tip interaction of “tip pressing” was performed near the helical dislocation of spirals. Before tip pressing, the sample bias and tunneling current were set to −1.30 V and −102 pA, respectively. The feed‐back of the tunneling current was turned off when the tip pressing was performed. Then, we directly reduced the tip height from the original tip position, and the descent of the tip was at least 2.5 nm for operations in this work. The sample bias was fixed during the pressing process. After the tip pressing, the tip was retracted and then reengaged for followed measurements. HVI were performed as conventional constant current imaging process. The sample bias and tunneling current were set to +4 V and +1 nA, respectively. The number of lines per frame for a 60 × 60 nm^2^ imaging region was at least 64 in the HVI process. All STM images in this paper were processed by the software named WSxM.^[^ ^34^ ^]^

## Conflict of Interest

The authors declare no conflict of interest.

## Supporting information

Supporting Information

## Data Availability

The data that support the findings of this study are available from the corresponding author upon reasonable request.
