# Supplementary material for: Strain Effects in Twisted Spiral Antimonene
Source: Adv Sci (Weinh). 2023 Apr 24;10(19):2301326. doi: 10.1002/advs.202301326 (PMC10323604; doi:10.1002/advs.202301326)
Supplement: Supplementary file 1 — Supporting Information [file ADVS-10-2301326-s001.pdf]

ADVANCED SCIENCE
Open Access

Supporting Information

for *Adv. Sci.*, DOI 10.1002/advs.202301326

Strain Effects in Twisted Spiral Antimonene

Ding-Ming Huang, Xu Wu, Kai Chang, Hao Hu, Ye-Liang Wang, H. Q. Xu* and Jian-Jun Zhang**

Supporting Information I:

The growth mechanism in this work is different from the previous study of spiral growth on non-Euclidean substrate surface.[9] Although the "step loops" on the Ge substrate are non-Euclidean structures, the inter-layer twist derived from this geometry is calculated as less than 1°, which is quite smaller than the value measured from the moiré pattern on spirals of 3°. The mechanism of step edge induced spiral growth [35] is also failed in this work. Antimonene is repelled to form helical dislocation, if there are only long parallel atomic steps present on the substrate (Fig. SI. 1a).

We speculate that the formation of helical dislocation is induced by strain accumulation inside the "step loop". The theoretical value of in-plane lattice constant of antimonene is 4.12 Å, which is about 3% larger than the Ge (111) surface. When the first layer antimonene grows and fills the "step loop", the lattice mismatch induced strain in antimonene neither relax at the edge due to constrain of "step loop" nor by forming undulation since we didn't observe it in the experiment, which is probably due to the relative strong interaction between antimonene and As/Ge(111) surface. Such large, accumulated compressive strain could induce novel structure formation. A possible way for the strain relaxation could be vacancy aggregation.[36] The Sb vacancies are tended to assemble into line structure under such large compressive strain and form a crack, as showed in Fig. SI. 1b. Further growth of the antimonene may lead to the step flow growth along one edge of the crack and formation of the spiral structure. The schematic diagram of spiral growth is illustrated in Fig. SI. 1c-g. Indeed, we found in the experiments that the density of the spiral is comparable to that of the "step loop", indicating that the spiral structure is induced by the surface "step loop". When we grow Sb on a flat As/Ge(111) surface (only low density intrinsic parallel atomic steps are presented on the substrate), we found that antimonene is tend to forming multilayers island but not spiral (Fig. SI. 1a), it is probably due to the strain relaxation induced by the free edge of the 2D antimonene island, which also support our proposal, that the growth of the spiral structure is induced by the strain accumulation of first layer antimonene in the "step loop".

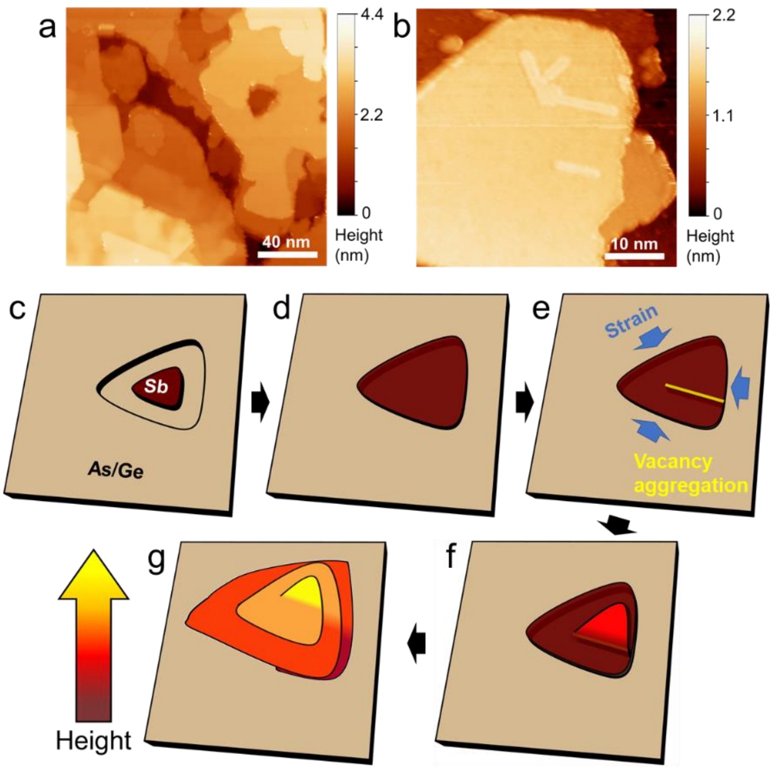

Figure SI. 1. Growth mechanism of the spiral antimonene. a) Multilayers islands of antimonene is formed when the substrate has no "step loop". b) Vacancy lines on antimonene. c~g) Schematic diagram of spiral growth. The compressive strain is accumulated as the antimonene flake expanding. Free edge relaxation is constrained by the "step loop", and vacancy lines formed. Helical dislocation is then formed on the vacancy line.

Supporting Information II

The Fig. 2e. in the paper main text is obtained by integrating the linecut profiles from the FFT images of QPI dI/dV maps. The profiles are recalibrated by normalizing the intensity of moiré points.

The energy dependence of the intra-band scattering vector is fitted to quadratic equation:

$$E = A * q^2 + B$$

where, E is the energy level. The wave number of scattering vector q is determined as the maximal momenta of the QPI peaks in FFT profile, and the error bar is the scale of pixel in FFT images of 0.02 nm^{-1}. The fitting curves for two different Γ-K directions are illustrated in Fig. SI. 2a. The coefficient B of these two fitting curves are determined as 470±5 meV and 459±9 meV, respectively. As we assume that the scattering is an intra-band scattering, the band minimum is equal to B. The band minimum is finally determined to be 465±10 meV.

The coefficient A is related to electron effective mass m_{eff}, which has the relation of:

$$m_{eff} = \frac{\hbar^2}{8A}$$

The m_{eff} in these two fitting curves are determined as 0.008 m_e and 0.005 m_e, respectively. An asymmetry of the electron scattering behavior is clearly observed.

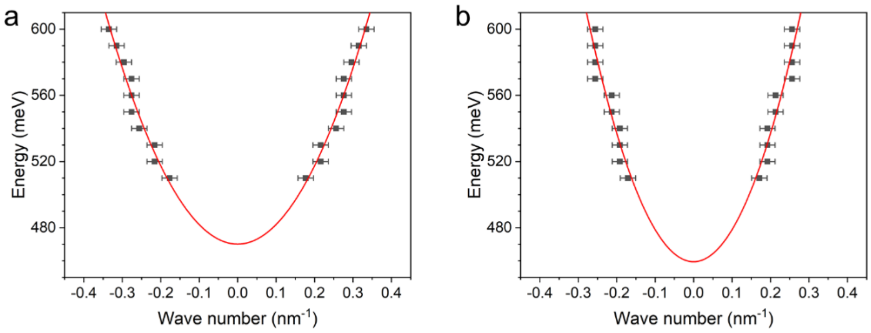

Figure SI. 2. Fitting curves of the energy dependence of the QPI scattering vector. a) and b) are fitting curves in two different Γ-K directions, respectively.

Supporting Information III

We put the original data of STM images on Fig. 3b of the paper main text, for intuitively demonstrate the twist angle. It should be noted that the true twist angle is not accordant to the dash line in Fig. 3b, owing to the measurement error from double-tip effect and scanning shift. The double-tip effect lows down the contrast of the step edge, and the scanning shift arises a tiny deformation on the image. In order to eliminate the error from double-tip effect, we use the reciprocal signal of the step edge in FFT image to define the normal direction of the edge, and the low contrast details of the step edge can no longer affect the determination of the step direction. We assume that all of step edges on the spiral are zigzag edges. The error from scanning shift can be eliminated, by recalibrating the zigzag edges in STM and FFT images. The recalibrated topographic image of the spiral and the corresponding FFT image are showed in Fig. SI. 3a and b, respectively.

In order to determine the tip pressing induced twist, the variation of intensity distribution along a line section in FFT images are measured, which is showed in Fig. SI. 3c. The measuring section line is illustrated by a white dashed line in Fig. SI. 3b. The twist induced by tip pressing can be calculated by the shifts of the peaks in the FFT profiles (Fig. SI. 3c). The tip pressing induced twist is eventually determined to be 1.0° ± 0.3°.

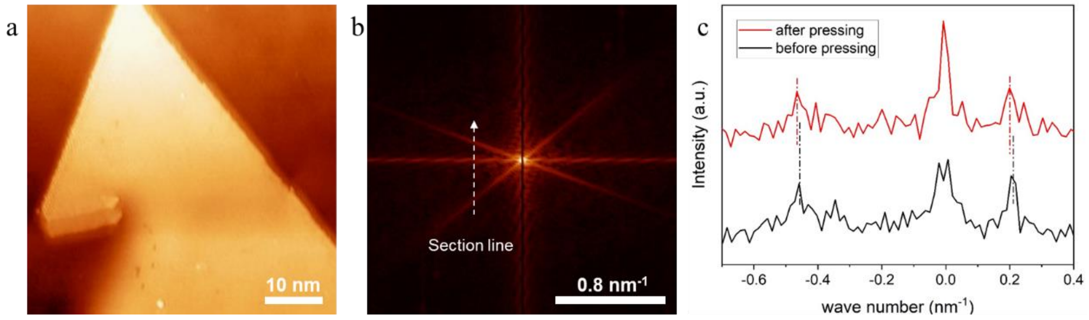

Figure SI. 3. Determination of the tip pressing induced twist. a) and b) Recalibrated STM image of the spiral and the FFT image, respectively. c) The section profiles along the white dashed line in b. Black and red curves are profiles before and after tip pressing, respectively.

Supporting Information IV

The current in tunnel junction is dependent to the sample voltage V and the barrier width W, by the relation of:

$$I \propto V\rho\, \mathrm{e}^{-2\frac{\sqrt{2\mathrm{m}_e \Phi}}{\hbar}W}$$

where, ρ is the density of states of tunneling electron, $\mathrm{m_e}$ is the mass of electron and Φ is the surface work function. The current I is exponential decayed with increasing of W, once V is fixed.

The work function Φ is characterized by I-Z spectra. The STM tip is grounded and the sample voltage is set at -1.3 V during measurements. The I-Z spectra is then fitted to the equation of:

$$I = \mathrm{A}\,\mathrm{e}^{-\mathrm{B}*Z}$$

The work function Φ is obtained from the tunnel-junction-apparent-barrier-height, which is decided to the exponential decay factor of B:

$$\Phi = \frac{\hbar^2}{8\mathrm{m}_e}\mathrm{B}^2$$

All of the I-Z spectra in this work exhibit well exponential relation, and an example is shown in Fig. SI. 4a.

Each work function data point in Fig. 4a of the paper main text is averaged from 6 groups of I-Z spectra, and the RMS error of the data points are about few hundreds of meV. An apparent change in the work function of a spiral is presented only after a deep "tip pressing", that the tip height is reduced by at least 2 nm. The work function is constant during I-Z measurement, as the tip height is changed within 0.25 nm. The non-negligible RMS error is not a measurement error caused by tip induced work function variation, but a systemic error.

dZ/dV spectra are measured to determine the work function variation with higher precision. Once the electric potential in the tunnel junction is approximated by a triangular shape. The energy levels of the field resonance states in the triangular potential are given by:

$$E_n = \Phi + \alpha_n F^{2/3}$$

Where, Φ is the surface work function, F is the electric field (which is a constant in a triangular potential), and α_n is the coefficient related to the quantum number n. [28,29]

The energy shift (ES) of the field resonance states between different materials is described by:

$$\Delta E_n = \Phi_1 - \Phi_2 + \alpha_n (F_1^{2/3} - F_2^{2/3})$$

In the condition of F_1=F_2, the ES of different resonance states are independent to the quantum number n, and the ES of all states are equal to the difference of the work function.

The "Gundlach oscillation peaks" in STM dZ/dV spectrum are caused by the field resonance states in tunnel junction. We can determine the work function variation by studying the ES of the "Gundlach oscillation peaks". Such method is generally applied to determine the work function in nano scale with precision of ± 10 meV. [37~40]

The dZ/dV spectra in this paper are obtained by numerical differentiation of the Z-V spectra.

Three sorts of dZ/dV spectra on a spiral antimonene are shown in Fig. SI. 4b, which are measured along three times of STM tip interactions. The ES of all peaks in the black and red curves are equal to 0.08±0.01 eV, corresponding to a work function variation of 0.08 eV after the tip interaction. The ES of the peaks in the red and green curves are inconsistent for different field resonance states. The inconsistence is caused by the change of the electric field F in tunnel junction, which may originate from a considerable variation of work function too (~1 eV scale). The dZ/dV spectroscopy is no longer available to detect the work function variation in such condition.

In summary, I-Z spectrum is efficient to detect the work function variation in the energy scale of 0.1~1 eV, with the systemic error of few hundreds of meV. In the other hand, dZ/dV spectrum is efficient to detect a work function variation in the energy scale of ~0.1 eV, with a high precision of ±10 meV. We must use both of two methods to demonstrate the tip induced work function variation, as which is presented in the paper main text.

It is worth noting that the tip induced work function variation is widely reproducible, but the variation rules for different spirals are not consistent. The work function of a spiral may increase after tip pressing, which is totally opposite to the data in Fig. 4a. Such difference between spirals is comprehensible, as the helical dislocation is essentially a disorder in the lattice, and each spiral has its own unique stacking order and original strain condition.

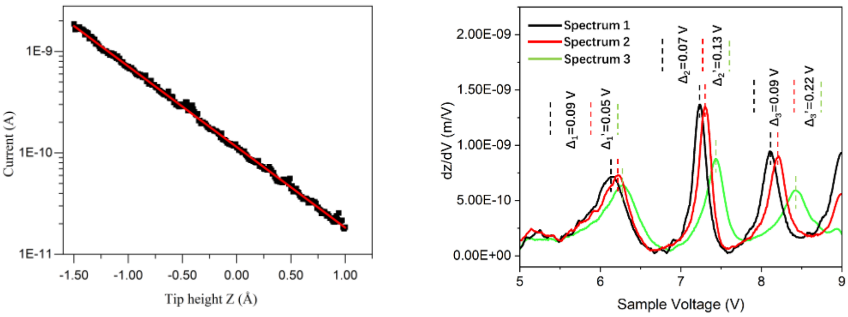

Figure SI. 4. Spectroscopy for work function determination. a) A representative I-Z spectrum in the logarithmic coordinates. b) The energy shift of the Gundlach oscillation peaks in three representative dZ/dV spectra.

Supporting Information V

In order to demonstrate the effect of inter-layer coupling to the work function variation, a "dual-helical spiral" is studied. The STM morphology of the "dual-helical spiral" is shown in Fig. SI. 5a, there are two helical dislocations in the spiral. The Burgers vectors of these two dislocations are opposite to each other. A "single-helical spiral" is free to twist under external interaction, and the helical dislocation is the twist axis. But the "dual-helical spiral" is unable to twist, because the twists around these two dislocations are inter-locked by each other (Fig. SI. 5b). As a consequence, in-plane distortion is relatively hampered on the "dual-helical spiral". The tip induced work function variation of a "dual-helical spiral" is shown in Fig. SI. 5c. The "tip pressing" is obviously able to change the work function, but the HVI is almost failed.

Here we propose a hypothesis to understand the above-mentioned phenomenon: The distortion induced by "tip pressing" consist of two parts, in-plane and inter-layer distortion, respectively. Both of them can be resided in a "single-helical spiral", especially the in-plane distortion is correlated to the inter-layer twist. However, only the distortion of inter-layer spacing can be resided in a "dual-helical spiral", as the twist is prohibited. The "single-helical spiral" is not as stable as the "dual-helical spiral" after STM tip pressing, as a result of the combination of distortions. The tip induced force in HVI is quite weaker than that in "tip pressing". The interaction of HVI is strong enough to relax the distortion in "single-helical spiral", but it is unable to relax the more stable distortion in "dual-helical spiral". That is why the HVI is failed to change the work function of the "dual-helical spiral" (Fig. SI. 5c).

Under such hypothesis, the work function variation caused by "tip pressing" in Fig. SI. 5c is originated from the distortion of inter-layer spacing.

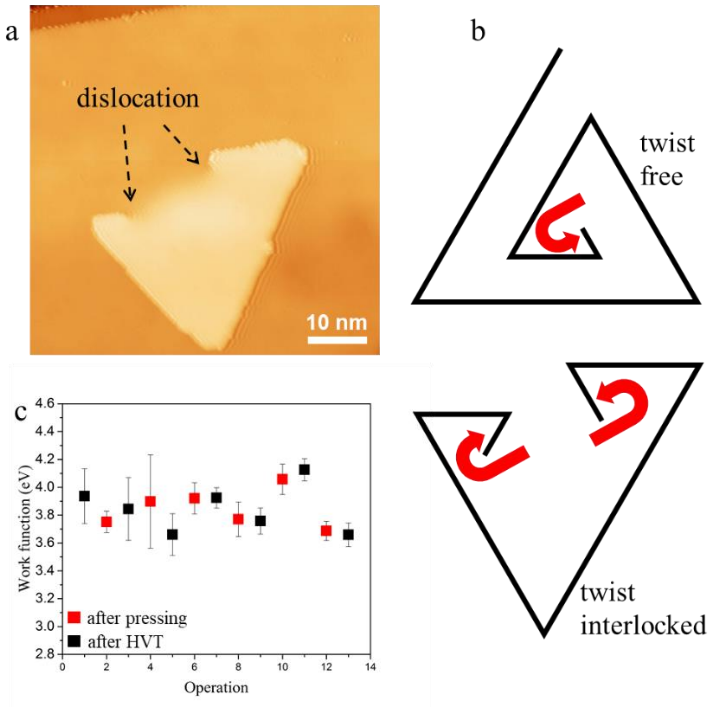

Figure SI. 5. Dual-helical spiral. a) STM topographic image of a "dual-helical spiral". b) Schematic diagram of the twist in "single-helical spiral" and "dual-helical spiral". c) Variation of the work function in a "dual-helical spiral".

Supporting Information VI

In order to verify that the DOS variation in the paper main text is caused by distortion, but not a measurement error from the local-electronic-states on moiré superlattice, the dI/dV line map on the moiré pattern is studied. The line map is shown in Fig. SI. 6a and the measuring line is illustrated by dashed line in Fig. SI. 6c. We can clearly observe that only a few states near the Fermi-level are location-relevant (marked by red triangle in Fig. SI. 6a). The broad peak between 0 mV and 200 mV is almost independent to the location. We speculate that the local-electronic-states are dependent to the local stacking rule on the moiré pattern,[11] and the broad peak in the line map is caused by a global surface state. The local work functions on the moiré pattern are also studied, and the dZ/dV spectra are shown in Fig. SI. 6b. The measuring positions are marked by dots in Fig. SI. 6c. The energy shift of the "Gundlach oscillation peaks" is within 30 meV, indicating that the location-relevant work function variation is smaller than 30 meV. In summary, the tip induced variations of DOS and work function are indeed structure-distortion-relevant, and are independent to the local-states on moiré superlattice.

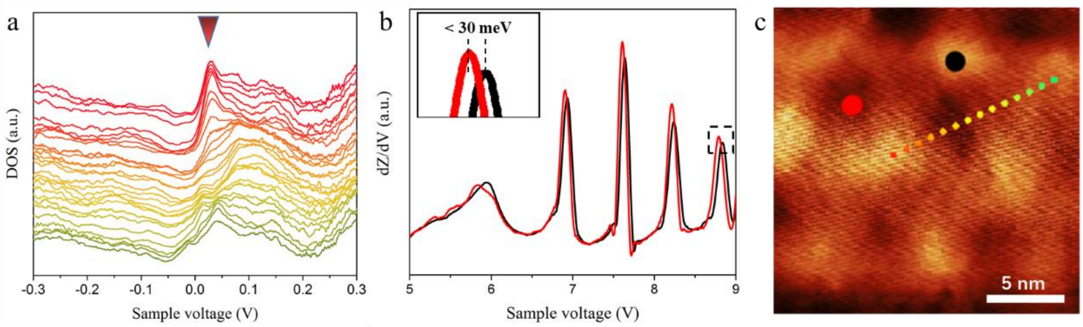

Figure SI. 6. Local-electronic states and local work functions on the moiré pattern. a) dI/dV line map on the moiré pattern. b) dZ/dV spectra on different positions. c) STM image of the moiré pattern. Measuring positions of the dI/dV line map and the dZ/dV spectra are marked by dashed line and dots, respectively.

Reference：

[35] Y. R. Chang, N. Higashitarumizu, H. Kawamoto, F. H. Chu, C. J. Lee, T. Nishimura, R. Xiang, W. H. Chang, S. Maruyama, K. Nagashio, *Chem. Mater.* **2021**, 33, 186.

[36] X. Lin, J. C. Lu, Y. Shao, Y. Y. Zhang, X. Wu, J. B. Pan, L. Gao, S. Y. Zhu, K. Qian, Y. F. Zhang, D. L. Bao, L. F. Li, Y. Q. Wang, Z. L. Liu, J. T. Sun, T. Lei, C. Liu, J. O. Wang, K. Ibrahim, D. N. Leonard, W. Zhou, H. M. Guo, Y. L. Wang, S. X. Du, S. T. Pantelides, H. J. Gao, *Nature Mater.* **2017**, 16, 717.

[37] C. L. Lin, S. M. Lu, W. B. Su, H. T. Shih, B. F. Wu, Y. D. Yao, C. S. Chang, T. T. Tsong, *Phys. Rev. Lett.* **2007**, 99, 216103.

[38] M. Pivetta, F. Patthey, M. Stenge, A. Baldereschi, W. D. Schneider, *Phys. Rev. B* **2005**, 72, 115404.

[39] C. Pauly, M. Grob, M. Pezzotta, M. Pratzer, M. Morgenstern, *Phys. Rev. B* **2010**, 81, 125446.

[40] K. Chang, J. W. D. Villanova, J. R. Ji, S. Das, F. Küster, S. Barraza-Lopez, P. Sessi, S. S. P. Parkin, *Adv. Mater.* **2021**, 33, 2102267.
